# FBXO5 acts as a novel prognostic biomarker for patients with cervical cancer

**DOI:** 10.3389/fcell.2023.1200197

**Published:** 2023-06-28

**Authors:** Shan Jiang, Jianfeng Zheng, Zhaolei Cui, Yanhong Li, Qiaoling Wu, Xintong Cai, Chaoqiang Zheng, Yang Sun

**Affiliations:** ^1^ Department of Gynecology, Clinical Oncology School of Fujian Medical University, Fujian Cancer Hospital, Fuzhou, China; ^2^ College of Integrative Medicine, Fujian University of Traditional Chinese Medicine, Fuzhou, China; ^3^ Laboratory of Biochemistry and Molecular Biology Research, Department of Clinical Laboratory, Clinical Oncology School of Fujian Medical University, Fujian Cancer Hospital, Fuzhou, China

**Keywords:** WGCNA, autophagy, cervical cancer, prognosis, immunity

## Abstract

**Background:** Cervical cancer (CC) remains one of the most common and deadly malignancies in women worldwide. *FBXO5*, a protein-coding gene, is highly expressed in a variety of primary tumors and promotes tumor progression, however, its role and prognostic value in CC remain largely unknown.

**Methods:** A key differential gene, *FBXO5*, was screened according to WGCNA based on immunohistochemical assays of clinical samples, multiple analyses of the Cancer Genome Atlas (TCGA) and Genotype-Tissue Expression (GTEx) databases, including survival analysis, tumor mutational burden, GO, KEGG, tumor immune infiltration, and chemotherapeutic drug sensitivity, to explore the expression and prognostic value of *FBXO5* in CC. The migration and invasiveness of cervical cancer cells following *FBXO5* knockdown and overexpression were examined using wound healing and transwell assays, and the viability of cancer cells was assessed using CCK8 and EdU assays.

**Results:** *FBXO5* was discovered to be substantially expressed in CC tissues using data from our CC cohort and the TCGA database, and a survival analysis indicated *FBXO5* as a predictive factor for poor overall survival in CC patients. *In vitro*, CC cells were more inclined to proliferate, migrate, and invade when *FBXO5* was upregulated as opposed to when it was knocked down.

## 1 Introduction

One of the most common female cancers in the world, cervical cancer can be surgically treated in early-stage patients and has limited efficacy in patients with intermediate to advanced disease, despite combined radiotherapy (Rahangdale et al., 2022; Ahmed et al., 2023). Death is primarily brought on by cancer’s metastasis and progression, giving patients a poor prognosis (Liontos et al., 2019; Coleman et al., 2021). Currently, the prognosis of cervical cancer is mainly assessed by squamous cell carcinoma antigen (SCC), however, this indicator does not change significantly in some patients with recurrence (Markovina et al., 2018; Shi et al., 2023). Finding additional prognostic indicators is, therefore, necessary to improve the prognosis of cervical cancer patients, as SCC alone is insufficient to determine the patient’s prognosis.

F-Box Protein 5 (*FBXO5*), also known as early mitotic inhibitory factor 1, is highly expressed in glioma, breast cancer, colorectal cancer, and hepatocellular carcinoma, and promotes tumor infiltration and metastasis (Zhao et al., 2013; Chao et al., 2014; Marzio et al., 2019; Wang et al., 2020). *FBXO5* acts as a regulator of anaphase-promoting complex/cyclosome (APC/C) activity during the mitotic and meiotic cell cycles regulator, enters the cell cycle by upregulating the expression of *FBXO5* mRNA, *APC/C* is responsible for the repeated elimination of mitotic cyclins to ensure cell division after each round of DNA replication. APC/C ubiquitin ligase destabilizes key regulators at predetermined points in the cycle (Di Fiore and Pines, 2007; Marzio et al., 2019). This leads to the conversion of *FBXO5* from an *APC/CCDH1* substrate to an inhibitor and the unidirectional inactivation of *APC/CCDH1*. *FBXO5* also interacts with EVI5, which accumulates early in G1 and induces premature degradation of *FBXO5*, leading to premature activation of *APC/C*, cell cycle disruption, over-replication of cell centrosomes and ultimately induction of the *Plk/SCF* pathway via early degradation of *FBXO5*, thereby disrupting mitosis (Cappell et al., 2018).

A highly conserved lysosomal breakdown system called autophagy is linked to the *in vivo* upkeep of cellular homeostasis. On the one hand, activated autophagy prevents tumor growth by several methods, including reducing oxidative stress and inhibiting genomic damage. On the other hand, in the face of numerous stress stimuli such as a lack of oxygen, energy, or growth factors, autophagy gives cancer cells the nutrition and energy they need to sustain their fundamental survival and metabolism (Deegan et al., 2013; Dikic and Elazar, 2018). Improving cancer cells’ capacity to survive in a deprived and hypoxic environment during the phase of fast tumor multiplication, especially in the later stages of tumor development promotes tumorigenesis. According to earlier research, autophagy can increase tumor growth by impairing immunity (Amaravadi et al., 2019). Considering that autophagy involves many different cellular processes, the molecular mechanisms of tumor development are extremely complex, and autophagic turbulence is one of the possible causes of tumor formation and development. As autophagy is a sensitive and complex multi-step regulatory process with a high degree of susceptibility, autophagy can be disrupted at any stage, which may lead to the development of the entire pathology (Levine and Kroemer, 2008). A recent study found that silencing genes associated with HPV16 enhanced cellular autophagy and senescence, and also had a significant inhibitory effect on the oncogenic process of cervical cancer. The link between adenosine triphosphatase family protein 3A (ATAD3A), an anti-autophagy factor, and HPV infection has been demonstrated in cervical cancer, where ATAD3A silencing increased the number of autophagic vesicles and significantly reduced drug resistance in cervical cancer cells, further demonstrating the importance of autophagy in HPV-mediated cancer development (Lang et al., 2020). Given the complex role of autophagy in cells, it is important to study how autophagy affects specific types of disease so that lesions can be detected early and effective therapeutic strategies can be developed to curb the development of cervical cancer. Therefore, further study is required to discover whether FBXO5 can speed up the growth of cervical cancer by inducing autophagy (Cui et al., 2019).

In this study, we found that *FBXO5* expression was elevated in cervical tissues and that *FBXO5* overexpression stimulated autophagy, which in turn promoted the proliferation and migration of cervical cancer cells. According to these findings, *FBXO5* may provide a therapeutic target for the treatment of cervical cancer.

## 2 Materials and methods

### 2.1 Data and analysis

*FBXO5*mRNA data from 306 tumors and 13 adjacent cervical tissues were downloaded from the Cancer Genome Atlas (TCGA) and the Gene Expression Volume Association database (https://gtexportal.org/home, GTEx). Single-cell analysis gene expression data from the Gene Expression Omnibus (GEO) database (http://www.ncbi.nlm.nih.gov/geo/) (GSE168652). For combinational analysis, the FPKM values were then changed to the million per kilobase (TPM) values for the transcripts. To lessen batch effects brought on by deviations outside the realm of biotechnology, Package R’s “SVA” uses the “ComBat” method (Johnson et al., 2007). Relevant clinical data files were downloaded, including tumor depth of infiltration, distant metastases, lymph node staging, survival time and survival status. The GeneCards database provided genes with an association with autophagy (https://www.genecards.org/).

### 2.2 Gene set enrichment analysis

The differentially expressed genes between the high and low FBXO5 groups were found using the R package “Limma” (adjusted *p*-value 0.01) (Ritchie et al., 2015). Molecular biological differences were taken into account when identifying differential genes in the high and low expression groups (|log2foldchange| > 0.5, adjusted *p*-value 0.05). For further investigation, their expression in all samples was isolated. To determine the biological functions and potential signaling pathways of *FBXO5*, the “clusterProfiler” package and the “org.Hs.e.g.db” package of R software were used to screen the relevant genes for GO and KEGG enrichment analysis was performed to explore the biological functions and signaling pathways (Kanehisa and Goto, 2000; Yu et al., 2012).

### 2.3 Immune microenvironment

The ImmuneScore, StromalScore, and EstimateScore were obtained by running the “ESTIMATE” package of the R software (Yoshihara et al., 2013), representing the immune infiltration score, the stromal cell infiltration score, and the composite score, which is a combination of the stromal cell score and the immune cell score, respectively. The composite score is a combination of the stromal and immune cell scores. The “limma” package in the R software was used to classify the *FBXO5* gene into high and low-expression groups and to determine whether the level of immune cell infiltration, stromal cell infiltration, and tumor purity differed between the high and low-expression groups, and the associated clinical features (Ritchie et al., 2015).

### 2.4 Mutation analysis

Somatic cell mutation information for cervical cancer was obtained from the TCGA database. Based on the median level of *FBXO5*, 306 tumor samples were divided into high and low-expression groups. The relationship between cervical cancer mutations and survival was analyzed by comparing the high and low groups using the “maftools” package (Mayakonda et al., 2018).

### 2.5 Cell culture and cell transfection

The human CC-related cell lines HeLa were purchased from Meisen (Zhejiang, China). Cells were cultured in Dulbecco’s modified Eagle medium (DMEM, Gibco, United States) supplemented with 10% fetal bovine serum (FBS, Gibco, Australia) at 37°C in an environment containing 5% CO_2_.

*FBXO5* knockdown and overexpression lentivirus was purchased from genechem (Shanghai, China) and cells (6 × 10 ^4^ cells/well) were inoculated into 6-well plates and incubated for 24 h after lentiviral infection. Twenty-4 hours after infection, cells were injected with puromycin (3 μg/mL) for 3 weeks to obtain a stably transfected cell line. Overexpression and knockdown efficiency of *FBXO5* in CC cell lines was verified using qRT-PCR.

### 2.6 CCK8 assay

Cells were inoculated at 4×10^3^ cells/well into 96-well plates, with 5 sub-wells per group. 10 μL CCK-8 (Bimake, Houston, Texas) was added to each well at 24, 48, 72, and 96 h respectively. After incubation at 37°C for 2 h, the absorbance values at 450 nm were measured by an enzyme marker.

### 2.7 EdU staining test

EdU staining was used to determine the proliferation of cervical cancer cells. Cells were inoculated at 1×10^5^ cells/well into 24-well plates and incubated with EdU (Beyotime, Shanghai, China) for 2 h. Cells were fixed in 4% paraformaldehyde for 10 min at room temperature.

### 2.8 Cell migration and invasion assays

For wound healing assays, the wound gap was recorded at 0, 12, or 24 h after scratching with a 0 μL pipette tip. Transwell assays were examined using an 8 mm well Transwell chamber (Corning, CA, United States).

### 2.9 Real-time quantitative PCR

Total RNA was isolated from the samples using the Promega Total RNA Extraction Kit (Shanghai, China). Using the Transcriptor First Strand cDNA Synthesis Kit (Shanghai, China), we reverse-transcribed total RNA after RNA extraction to create cDNA. SuperReal PreMix Plus from Tiangen Biotech (Beijing, China) was used to determine the expression levels of *FBXO5*. In Supplementary Table S1, the primer sequences are displayed.

### 2.10 PI staining for cell cycle detection

Cells in the logarithmic growth phase were inoculated at 4×10^5^/well in 6-well plates and left overnight. After wall mounting, cells were digested with trypsin. After centrifugation, the cells were fixed in 70% ethanol overnight, centrifuged at 2000 r/min for 5 min before use, 70% ethanol was discarded, washed once with pre-cooled PBS, suspended with 0.5 mL RNase and then bathed in water at 37°C for 30 min, and finally added PI staining for 30 min in water at 37°C, filtered through a 300 mesh sieve and tested on the machine.

### 2.11 Western blot

The BCA protein concentration assay kit was used to assess the total protein content of CC cells. 20 μg of protein loading volume were electrophoretically separated on 10% SDS polyacrylamide gel, transferred to PVDF membranes, and sealed with TBST solution containing 5% BSA at room temperature. Following that, the membrane was incubated at 4°C for an overnight period with the diluted antibodies LC3, Beclin1, P62, and beta-actin (1:2000, proteintech, Wuhan, China) respectively. The samples were then developed using enhanced chemiluminescence after being re-incubated with goat anti-rabbit IgG antibody secondary antibody (1:5000) at room temperature for 1 h.

### 2.12 Immunohistochemistry

Tissue microarrays (HUteS136Su01) of CC specimens were obtained from Shanghai Outdo Biotech Company (http://www.superchip.com.cn/, Shanghai, China). To verify *FBXO5* expression in CC. Immunohistochemistry was then performed using staining cycles as shown below. Sections were washed and deparaffinized with xylene, 100% ethanol, 95% ethanol, 80% ethanol, and phosphate buffer, and endogenous peroxidase activity was blocked with 3% hydrogen peroxide. Non-specific antigenic epitopes were blocked by incubation with 10% goat serum in PBS for 1 h at room temperature. Tissue sections were incubated overnight at 4°C with primary monoclonal rabbit *anti-FBXO5* antibody at a dilution of 1:500 and then incubated for 1 h at room temperature with horseradish peroxidase-conjugated goat anti-rabbit secondary antibody. A total of 110 samples were collected by removing incomplete tissue sections and samples that lacked clinical information. Staining intensity score: unstained 0, light yellow 1, light brown 2, dark brown 3. The proportion of positive staining cells: less than 5% of positive cells count 0 points, 5% -25% of positive cells count 1 point, 25% -50% of positive cells count 2 points, and ≥50% of positive cells count 3 points. Each section was randomly selected to observe different representative areas, and a total of 5 fields of view were observed under high magnification. The total score was obtained by multiplying the staining intensity with the percentage of positive cells, and the average was taken. A total score greater than or equal to 3 is classified as a high expression, and a total score less than 3 is classified as a low expression.

### 2.13 Statistical analysis

The statistical analysis was performed using the R program (version 4.1.3). Statistical significance was defined as a two-tailed *p*-value of 0.05.

## 3 Results

### 3.1 Identification of key gene *FBXO5*

**FIGURE 1:**
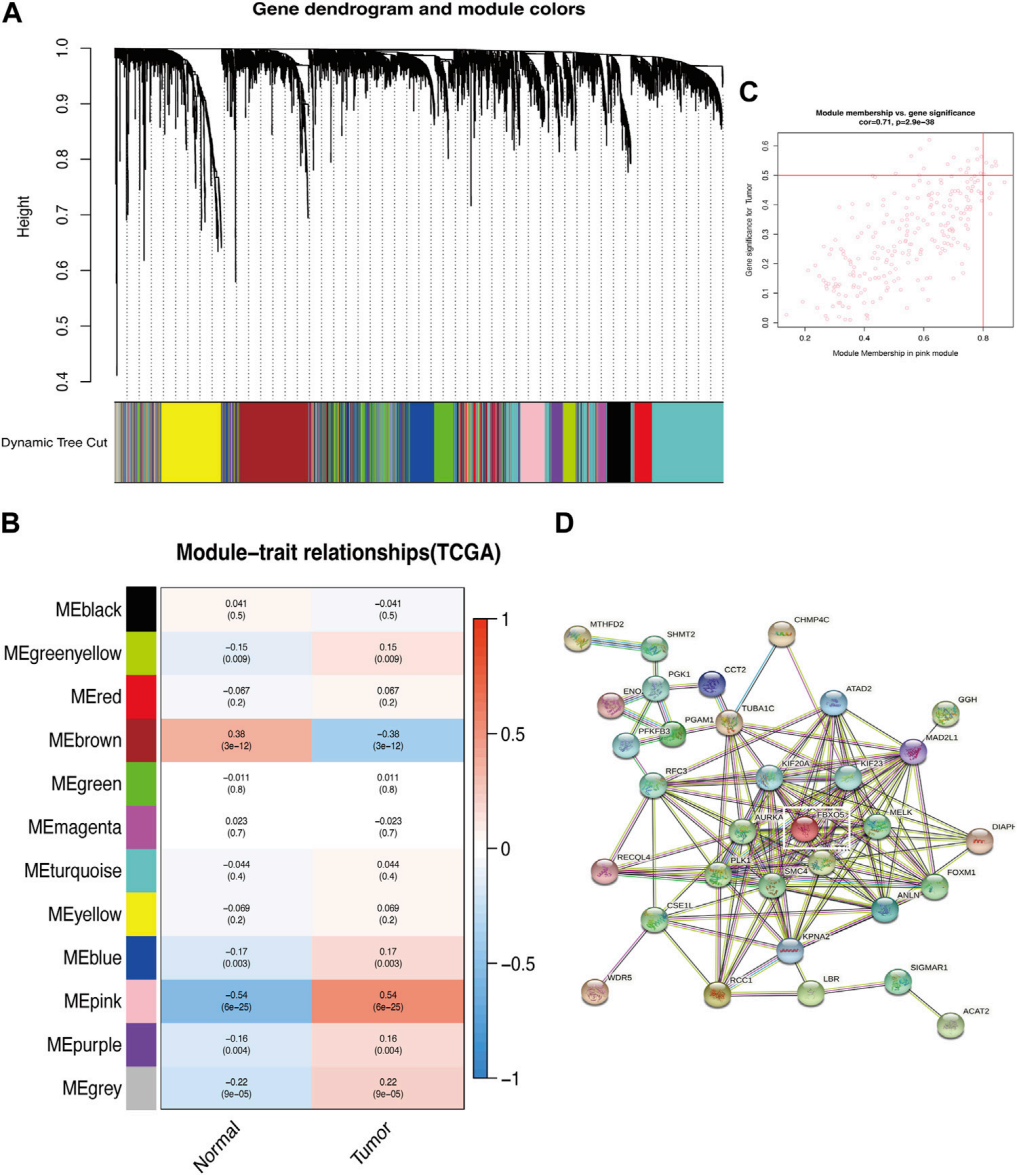
Identification of key modules of autophagy-related genes. **(A)** Gene clustering associated with autophagy-related genes. **(B)** Heatmap shows the relationship between gene modules and the normal/tumor type. Gene clustering associated with autophagy. **(C)** Correlation between the chosen module’s membership and gene importance. **(D)** Correlation between gene importance and the membership of the chosen module.

The genes associated with autophagy were correlated with the phenotypes using the WGCNA module, which demonstrated that the genes with extremely low expression were eliminated and the values satisfied the screening criterion at an ideal threshold of 10. The Pink module revealed the most significant link with “Tumor” (*p* = 6e-25, *r* = 0.54) and six out of the twelve modules had statistically significant differences (*p* < 0.05). With “Tumor” in the pink module (Figures 1A–C; Supplementary Figrues S1A, B). The “Pink” module’s PPI protein interaction network was built using the STRING online database, and the significant gene PDIA3 was obtained by excluding the autophagy-related genes previously demonstrated to be connected with cervical cancer patients’ prognosis (Figure 1D).

### 3.2 Expression of *FBXO5* in CC patients based on TCGA databases and function enrichment

**FIGURE 2:**
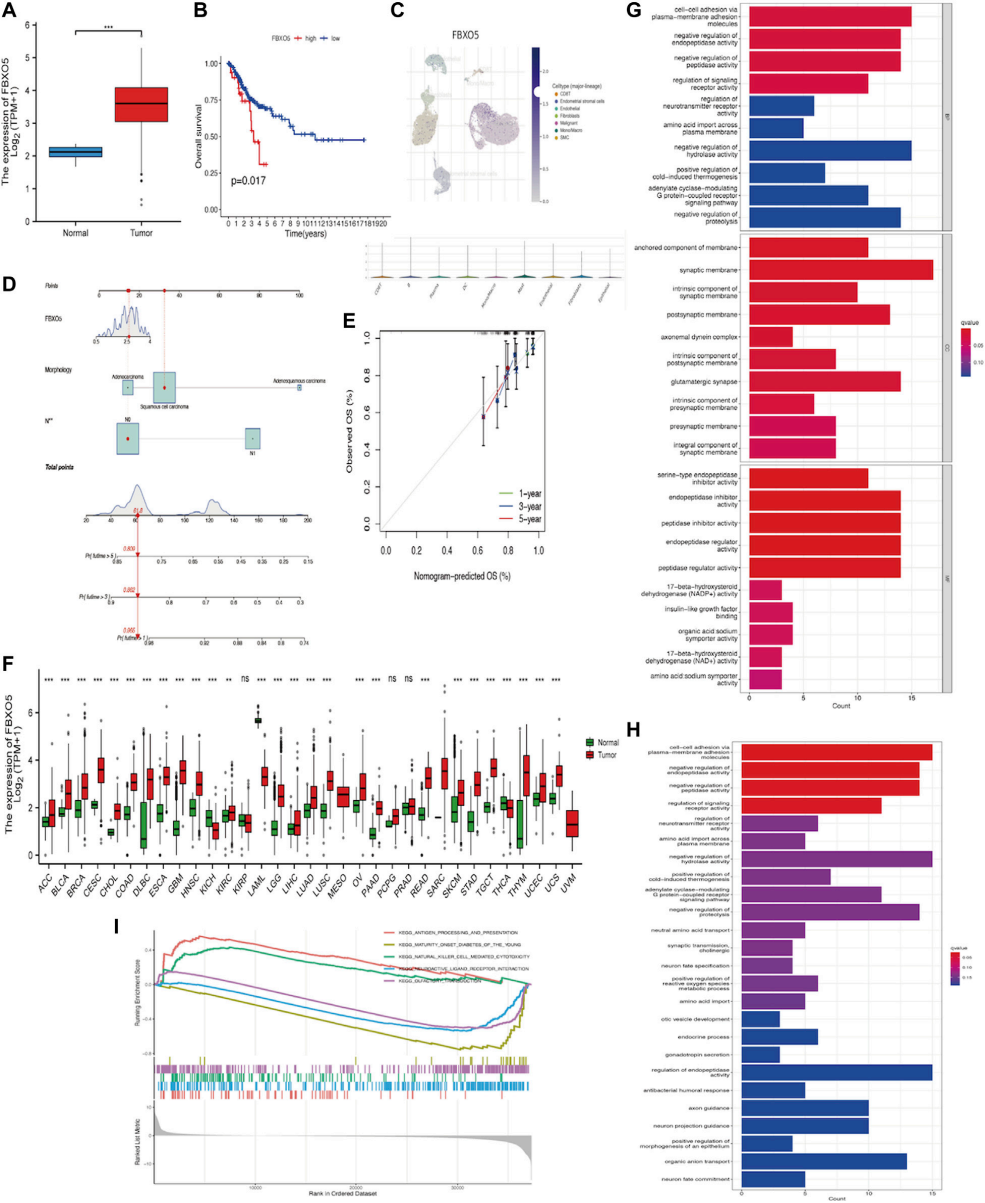
Expression of *FBXO5* in cancer **(A)** Expression of *FBXO5* in cervical cancer and normal tissues. **(B)** Analysis of the relationship between *FBXO5* and overall survival (OS) of cervical cancer patients in the TCGA dataset. **(C)** UMAP visualization of *FBXO5* in significant cervical cancer cell subpopulations (GSE168652). **(D)** The nomogram was constructed using risk factors. **(E)** Calibration plot of a nomogram for predicting overall survival in CC patients at 1 year, 3 years, and 5 years. **(F)** Pan-cancer *FBXO5* expression. **(G)** Bar graph of GO enrichment of *FBXO5*. **(H)** Bar graph of KEGG enrichment of *FBXO5*. **(I)** Enrichment of GESA pathway of *FBXO5*.

In cervical cancer tissues compared to normal tissues, *FBXO5* expression was higher (*p* < 0.001) (Figure 2A). Based on median *FBXO5* expression, patients were separated into high and low-expression groups, the high-expression group had worse survival rates (*p* = 0.017 Figure 2B). *FBXO5* was more broadly distributed in malignant cells, according to a single-cell analysis of expression in the tumor microenvironment, which included immune cells, stromal cells, malignant cells, and functional cells (Figure 2C). Lymph node staging and morphology are used clinically to create a nomogram, which can be used to forecast a patient’s prognosis more specifically (Figures 2D, E). Except renal papillary cell carcinoma, pheochromocytoma, paraganglioma, and prostate adenocarcinoma, *FBXO5* was expressed in a higher range of malignant tumors (Figure 2F). We explored the possible mechanisms of *FBXO5* in cervical cancer through Gene Ontology (GO), Kyoto Encyclopedia of Genes and Genomes (KEGG) and Gene Set Enrichment Analysis (GESA). GO and KEGG revealed biological processes focused on signaling actions such as cell-cell adhesion via plasma-membrane adhesion molecules, synaptic membrane and regulation of endopeptidase activity (Figures 2G, H), and GESA enrichment in cell cycle and PPAR signaling pathways (Figure 2I).

### 3.3 Based on our CC cohort, the increased expression and clinical relevance of *FBXO5* in CC were confirmed

**FIGURE 3:**
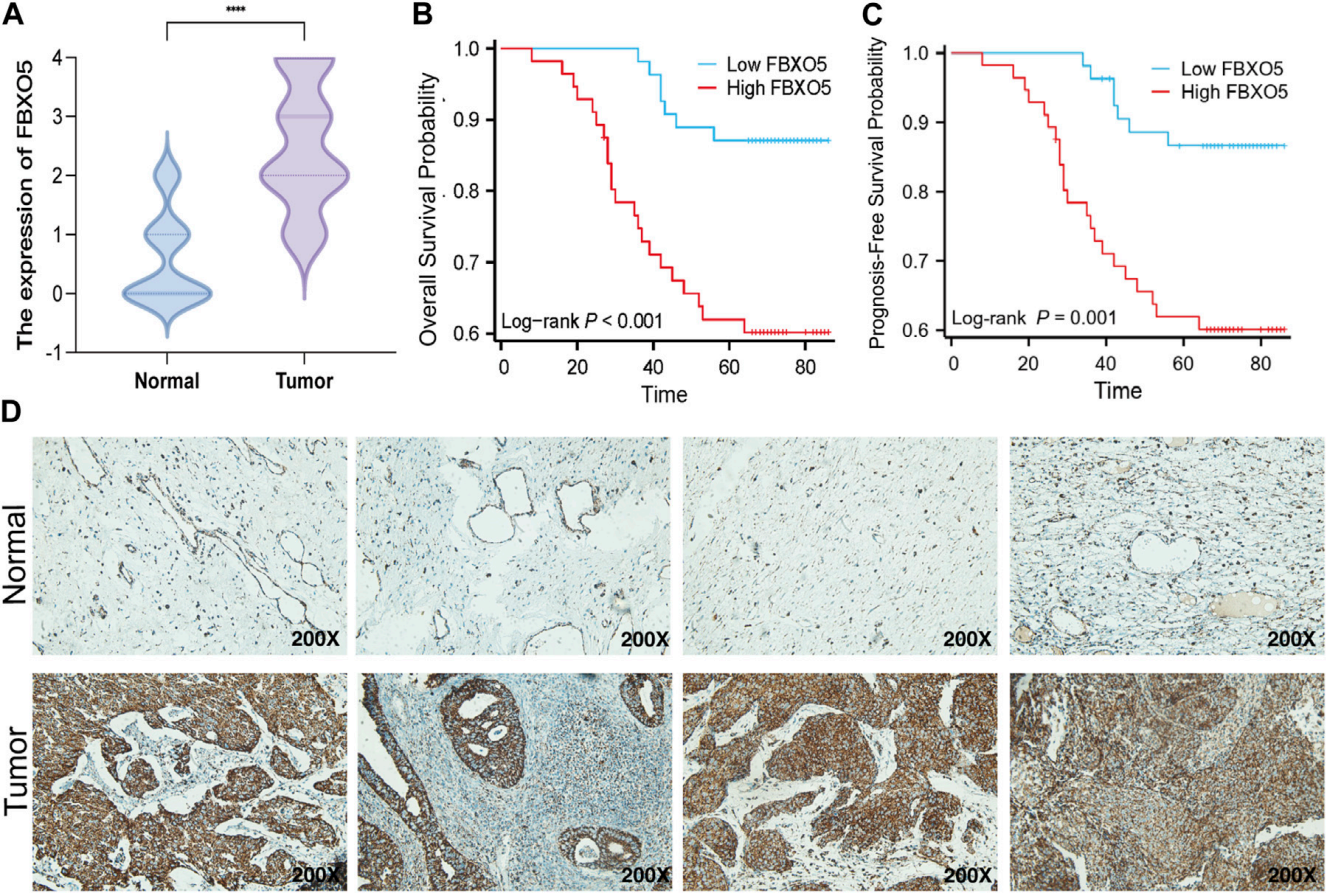
Expression of *FBXO5* in our CC cohort **(A)** *FBXO5* is differentially expressed in cervical cancer and normal tissue. **(B)** Analysis of the relationship between *FBXO5* and overall survival (OS) of cervical cancer patients. **(C)** Analysis of the relationship between *FBXO5* and progression-free survival (PFS) of cervical cancer patients. **(D)** *FBXO5* protein expression in normal cervical tissue samples and CC tissue samples.

Immunohistochemistry was performed on 135 cervical tumors and matched para-cancer samples to further establish the degree of *FBXO5* expression in cervical cancer (Figure 3D). In concordance with the TCGA findings, the comparison results showed that *FBXO5* was strongly expressed in cancer tissues and differently expressed in paraneoplastic samples (Figure 3A). Immunohistochemistry was used to classify the patients into two groups: high expression (*n* = 54) and low expression (*n* = 56). The expression level of the *FBXO5* protein linked with the patient’s pathological grade (*p* = 0.035) and N stage (*p* = 0.048), although clinical variables such as pathologic stage, age, and morphology did not (*p* > 0.05, Table 1), according to a correlation analysis of clinical data. Patients with high *FBXO5* expression had shorter overall survival and progression-free survival (PFS) than those with low expression, with a statistically significant difference (*p* < 0.001, *p* = 0.001), using the Kaplan-Meier method for survival prognosis analysis of cervical cancer follow-up data (Figures 3B, C).

**TABLE 1:** The correlation between clinicopathological data and *FBXO5* expression.

Characteristics	Low FBXO5	High FBXO5	*p*-value
n	54	56	
T stage, n (%)			0.037
T1	37 (33.6%)	25 (22.7%)	
T2	12 (10.9%)	17 (15.5%)	
T3	5 (4.5%)	13 (11.8%)	
T4	0 (0%)	1 (0.9%)	
N stage, n (%)			0.048
N0	49 (44.5%)	43 (39.1%)	
N1	5 (4.5%)	13 (11.8%)	
Pathologic stage, n (%)			0.557
Ⅲ	39 (41.9%)	40 (43%)	
Ⅱ	5 (5.4%)	3 (3.2%)	
Ⅰ	2 (2.2%)	4 (4.3%)	
Age, median (IQR)	45 (40, 48)	47 (42, 56.5)	0.071
Morphology, n (%)			0.765
Squamous cell carcinoma	50 (45.5%)	52 (47.3%)	
Adenocarcinoma	2 (1.8%)	1 (0.9%)	
Adenosquamous carcinoma	2 (1.8%)	3 (2.7%)	

### 3.4 Mutational analysis of *FBXO5* gene expression

**FIGURE 4:**
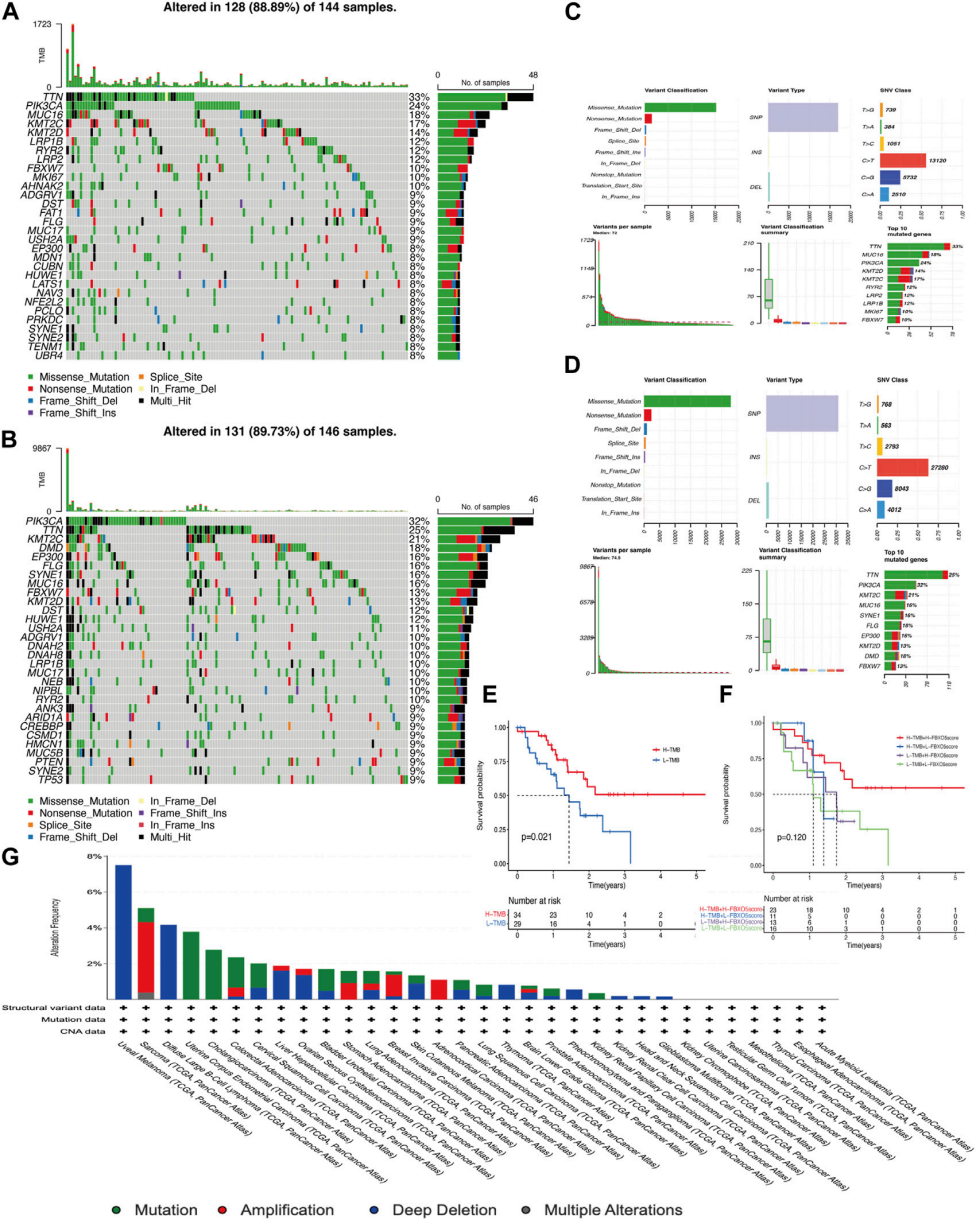
Mutational analysis **(A, C)** *FBXO5* high expression in patients with mutations. **(B, D)** *FBXO5* low expression in patients with mutations. **(E)** The Kaplan-Meier curves for the groups with low and high TMB are shown. **(F)** The Kaplan-Meier analysis curves for the patients were divided into groups based on TMB and *FBXO5* expression. **(G)** Analysis of the *FBXO5* mutation in pancytopenia.

The overall mutations in the groups with low and high *FBXO5* expression are shown in Supplementary Figures S1A, B. In addition, the majority of mutant genes communicate with one another (Supplementary Figures S1C, D). In both groups, the genes TTN, PIK3CA, KMT2C, and MUC16 were more frequently mutated. The mutation rate was higher in the *FBXO5* high-expression patients, the PIK3CA gene was mutated in more patients in this group, and TTN was the most abundant mutated gene (Figures 4A–D). The prognosis of the high tumor mutational burden (TMB) score group was worse than that of the low score group, and the prognosis of patients in the high TMB score group combined with high *FBXO5* expression was inferior to that of patients in the low TMB score group combined with low *FBXO5* expression, according to a survival analysis using Kaplan-Meier curves (Figures 4E, F). The mutation analysis of *FBXO5* in pan-cancer is shown in Figure 4G, with cervical cancer showing the majority of mutation and deep deletion.

### 3.5 The relationship between immune infiltration and *FBXO5*

**FIGURE 5:**
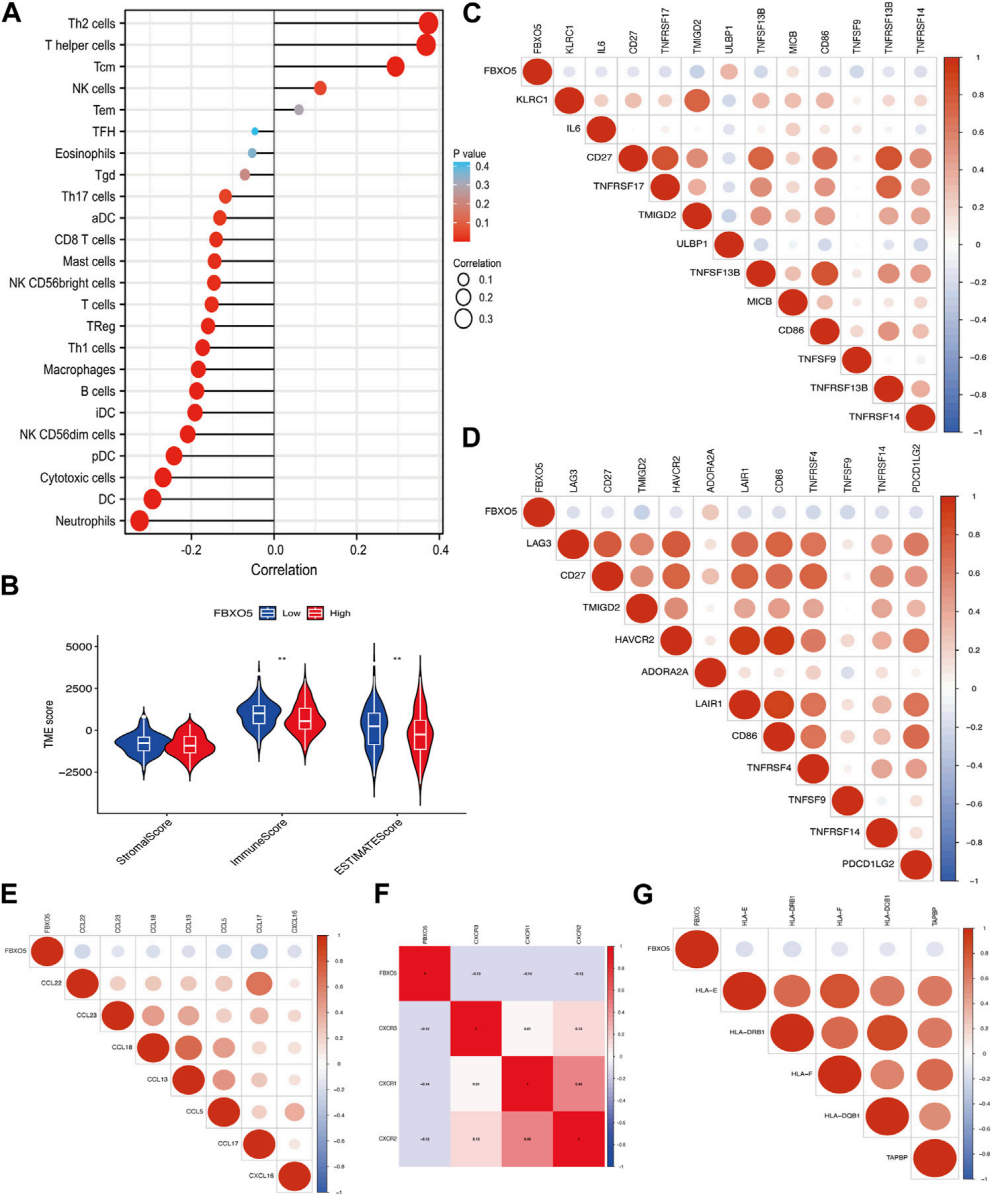
The relationship between immune infiltration and *FBXO5*. **(A)** Analysis of the correlation between *FBXO5* and immune cells. **(B)** Differential analysis of tumor microenvironment scores between the *FBXO5* high and low expression groups. **(C–G)** The relationship between *FBXO5* and immunomodulators: immunostimulants **(C)**, checkpoints **(D)**, chemokines **(E)**, receptors **(F)** and MHCs **(G)**.

*FBXO5* is associated with a variety of immune cells in cervical cancer, including Th2 cells, T helper cells, cytotoxic cells, dendritic cells, and neutrophils (Figure 5A). The *FBXO5* high expression group had different ImmuneScore and ESTIMATEScore results from the low expression group (*p* < 0.01), and the high expression group performed lower on the tumor immune microenvironment (TME) score than the low expression group (Figure 5B). The relationship between *FBXO5* and immunomodulators (chemokines, receptors, MHCs, immunostimulants, and checkpoints) was analyzed in order to further understand the impact of *FBXO5* on tumor immunity (Figures 5C–G).

### 3.6 Differences in immunomodulatory drug sensitivity

**FIGURE 6:**
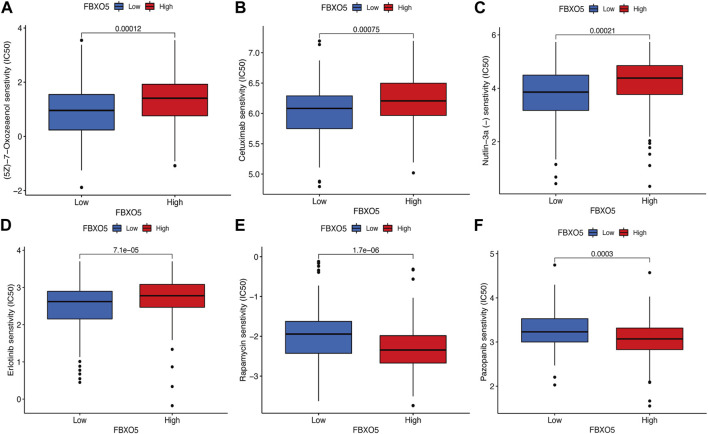
Differences in immunomodulatory drug sensitivity. **(A–F)** Differences in immunomodulatory drug sensitivity between the *FBXO5* high and low expression groups. (5Z)-7-Oxozeaenol **(A)**, Cetuximab **(B)**, Nutlin-3a **(C)**, Erlotinib **(D)**, Rapamycin**(E)** and Pazopanib **(F)**.

In order to evaluate the value of *FBXO5* for chemotherapeutic effectiveness, we examined the difference in sensitivity to frequently used chemotherapeutic drugs in CC between the high and low expression groups. Patients with high *FBXO5* expression were less responsive to Rapamycin and Pazopanib and more sensitive to (5Z)-7-Oxozeaenol, Cetuximab, Nutlin-3a, and Erlotinib (Figures 6A–F).

### 3.8 *FBXO5* promotes cervical cancer cells’ invasion and migration

**FIGURE 7:**
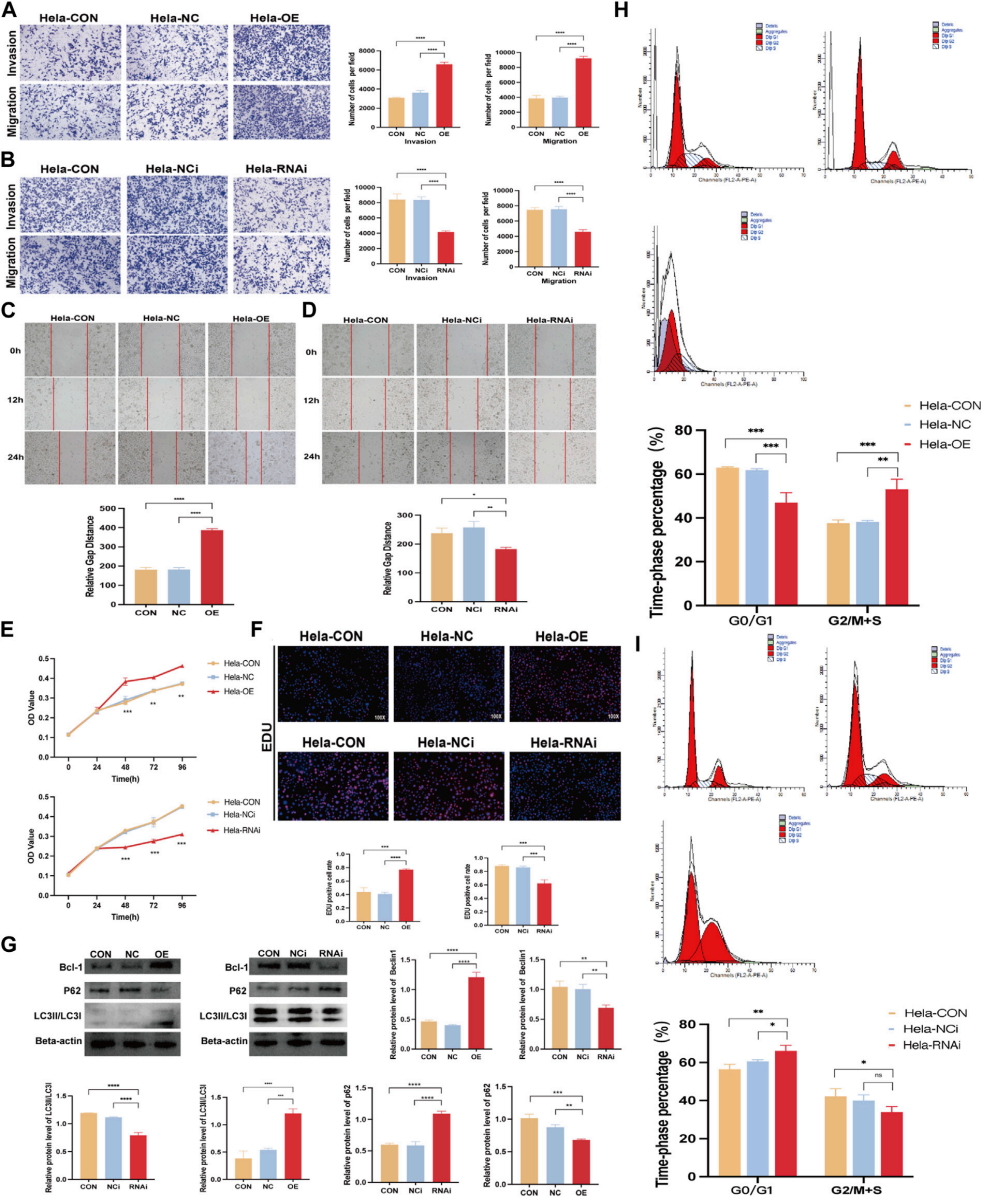
*FBXO5* promotes cervical cancer cells’ invasion and migration. **(A, B)** Transwell assays were used to evaluate the ability of HeLa cells to invade and migrate after *FBXO5* was overexpressed **(A)** and knocked down **(B)** for 48 h **(C, D)** Wound healing assays were used to evaluate the ability of HeLa cells to invade and migrate after *FBXO5* was overexpressed **(E)** and knocked down **(D)** for 72 h. **(A)** According to the CCK-8 assay, *FBXO5* upregulation encouraged Hela growth while *FBXO5* downregulation prevented it. **(F)** Percentage of Hela positive cells for EdU staining (×100). **(G)** Western blot was used to assess the levels of Bcl-1, LC3, and p62 protein expression in cells. **(H)** Detection of cell cycles in cervical cancer cells transfected with *FBXO5* (+) virus. **(I)** Detection of cell cycles in cervical cancer cells transfected with *sh FBXO5* virus.

We transfected *FBXO5* overexpression and knockdown virus in Hela and used qRT-PCR to assess the efficacy of *FBXO5* transfection in order to better research the function of *FBXO5* in cervical cancer (Supplementary Figures S1E, F; Table 1). As seen in the image, *sh FBXO5* transfected cancer cells exhibited much less capacity to invade and migrate than the control (Figures 7A, B). Consistent with the transwell assays results, the ability of *sh FBXO5*-transfected cancer cells to migrate was inhibited in wound healing assays (Figures 7C, D). The CCK8 assay results demonstrated that *FBXO5* knocked down cells drastically reduced the viability and proliferation of Hela cells (Figure 7E). The *FBXO5* overexpression group had noticeably more cells in a proliferative state than the knockdown group, according to the EdU assay (Figure 7F). To assess the effect of *FBXO5* on autophagy in cervical cancer, we examined the autophagy protein expression of the cells. According to the data, cells overexpressing *FBXO5* had higher levels of Bcl-1 and LC3 and lower levels of P62 expression when compared to the control group (Figure 7G). While the proportion of G2/M + S phase cells increased in the *FBXO5* group compared to the control group at a highly significant level, the number of G0/G1 phase cells was significantly lower in the transfected group than the control group (Figure 7H). In parallel, the opposing effect of *FBXO5* gene knockdown on the cell cycle was confirmed (Figure 7I), demonstrating that the expression of *FBXO5* would encourage cervical cancer cells to be halted in the G2/M + S phase.

## 4 Discussion

One of the most prevalent cancers in women, cervical cancer poses a significant risk to one’s health in terms of incidence and mortality (Sung et al., 2021). Autophagy plays a significant part in the development of cervical cancer, just like it does in other tumors (Wu et al., 2019; You et al., 2019; Chen et al., 2022). Histones, which activate autophagy in cervical cancer cells, or chemicals, which support the body’s immune response by facilitating macrophage phagocytosis through autophagy, both increase cancer growth (Orfanelli et al., 2014; Yang et al., 2021; Sun et al., 2022). More and more autophagy-related genes have been found to affect the growth and development of CC (Mattoscio et al., 2018; Yang et al., 2021). We discovered *FBXO5*, a crucial cervical cancer gene, using the WGCNA method, and discovered for the first time that *FBXO5* can affect the growth of cervical cancer by enhancing autophagy. We discovered that *FBXO5* expression was elevated in cervical cancer and adversely linked with patient prognosis using public data and our own cohort analysis. Moreover, we discovered that a nomogram built from *FBXO5* expression specifically predicted patient survival. Patients’ prognoses were also improved by higher TMB and *FBXO5* expression. According to previous research, patients with a higher TMB have a stronger capacity to produce neoantigens and a larger tumor immunogenic response, which makes them more likely to respond favorably to immunotherapy (Galvano et al., 2021; Zheng et al., 2022). *FBXO5* may be used as a prognostic indicator for tumor treatment in patients with CC by targeted therapy.

Many studies have previously found connections between the cell cycle and autophagy. For instance, the most important kinase in the cell cycle, *CDK1*, is phosphorylated to control the initiation protein *ULK1/ATG13*, which in turn promotes mitotic autophagy and cell cycle progression (Li et al., 2020).

The *FBXO5* gene was first discovered in extracts from the African clawed toad as a cell cycle protein that prevents the anaphase promoting complex (APC) from being activated by binding to cytokinesis cycle proteins, accumulating cytokine B, and advancing mitosis into M phase (Min et al., 2013; Zhao et al., 2013) As a critical regulator of mitosis, *FBXO5* prevents APC ubiquitination and encourages mitosis into the S phase. Aberrant *FBXO5* expression affects chromosome stability and regular cell division (Margottin-Goguet et al., 2003). In contrast, polyploidy production and chromosome instability are significant contributors to carcinogenesis and significant markers of malignancies, while normal cell division and chromosome stability are key requirements for the maintenance of normal bodily function (Liu et al., 2013). Biological functional enrichment analysis results also suggest that *FBXO5* is involved in cell cycle roles in patients with cervical cancer.

An analysis between *FBXO5* and immune infiltration was used to determine whether *FBXO5* can be used as a target for immunotherapy in patients with cervical cancer. The risk *FBXO5* was negatively correlated with TME scores, indicating that patients with fewer side effects by targeting this molecule have a stronger anti-tumor immune response, a greater potential to benefit from immunotherapy, and a longer survival time (Ji et al., 2012; Sanmamed and Chen, 2018). Many patients with malignant tumors now have hope of survival thanks to tumor immunotherapy, which uses immunological checkpoints as its focal site of action and extends survival times for patients with advanced stages (Brower, 2015). As tumors develop, several components of the innate and adaptive immune system in the tumor microenvironment are immunosuppressive, contributing to immune evasion of the tumor and resistance to checkpoint inhibitors. Several immunological checkpoints were negatively connected with *FBXO5*, which was also highly correlated with its closely related T-cells (Fan et al., 2021; Gao et al., 2023). The effector T-cells encourage tumor cell death, and their release of lysis factors is essential for the development of anti-tumor immunity (Jiang et al., 2018). Immune checkpoints will mount an anti-cancer immune response at an early stage to avoid overaction in the early stages of cancer, but this allows cancer to escape the immune system (Li et al., 2023). By utilizing targeted therapy in the early stages and combining it with the appropriate chemotherapeutic agents, we can stimulate the body’s immune system and increase the number of patients who benefit from cervical cancer treatment options.

By comparing our cohort with data from three public databases, this study was the first to validate the expression levels of *FBXO5* in CC and their effect on patient prognosis. As a result, it might develop into a stand-alone predictive risk factor and be used as a novel marker for the clinical diagnosis and prognosis of cervical cancer, supporting doctors in developing more patient-centered treatment regimens. In the future, additional long-term follow-up studies will be needed, along with the validation of our research utilizing a large number of normal samples and tumor samples. The results of this study also demand further investigation, such as animal and cell investigations, in order to more thoroughly validate the mechanism of FBXO5 promoting CC.

## 5 Conclusion

Cell cycle-related gene *FBXO5* induces autophagy in cervical cancer cells and is substantially expressed in CC. It is also gradually linked to poor patient outcomes. Our research fills in the gap regarding *FBXO5* in cervical cancer and points us that CC patients may be able to use it as a possible predictive biomarker.

## Data Availability

Publicly available datasets were analyzed in this study. This data can be found here: https://portal.gdc.cancer.gov/ https://www.ncbi.nlm.nih.gov/geo/.

## Abbreviations

APC, anaphase promoting complex; APC/C, anaphase-promoting complex/cyclosome; CC, cervical cancer; FBXO5, F-Box Protein 5; GEO, The Gene Expression Omnibus; GO, Gene Ontology; GSEA, Gene Set Enrichment Analysis; GTEx, Genotype-Tissue Expression; K- M, Kaplan-Meier; KEGG, Kyoto Encyclopedia of Genes and Genomes; OS, overall survival; PI, propidium iodide; SCC, squamous cell carcinoma antigen; TCGA, The Tumor Genome Atlas; TMB, tumor mutational burden; TME, Tumor microenvironment.
