# Supplementary material for: FBXO5 acts as a novel prognostic biomarker for patients with cervical cancer
Source: Front Cell Dev Biol. 2023 Jun 28;11:1200197. doi: 10.3389/fcell.2023.1200197 (PMC10338834; doi:10.3389/fcell.2023.1200197)

**Supplementary Material**

Supplementary Figure 1. （A-B）Estimation of the soft thresholding value for a scale-free co-expression network.（C-D）Correlation analysis of mutant genes（E-F）The effectiveness of FBXO5 transfection was assessed using the CT value from qRT-PCR.


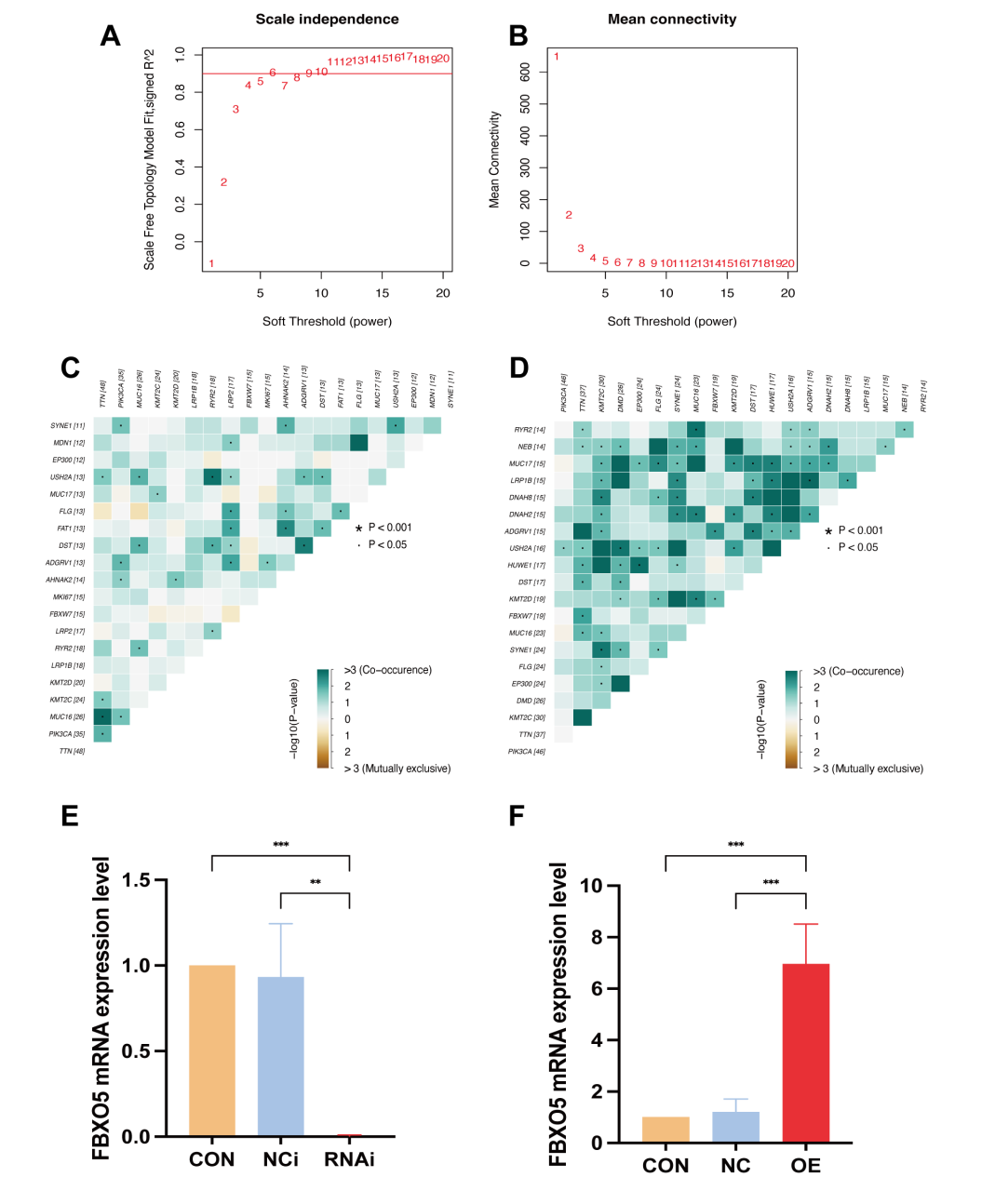


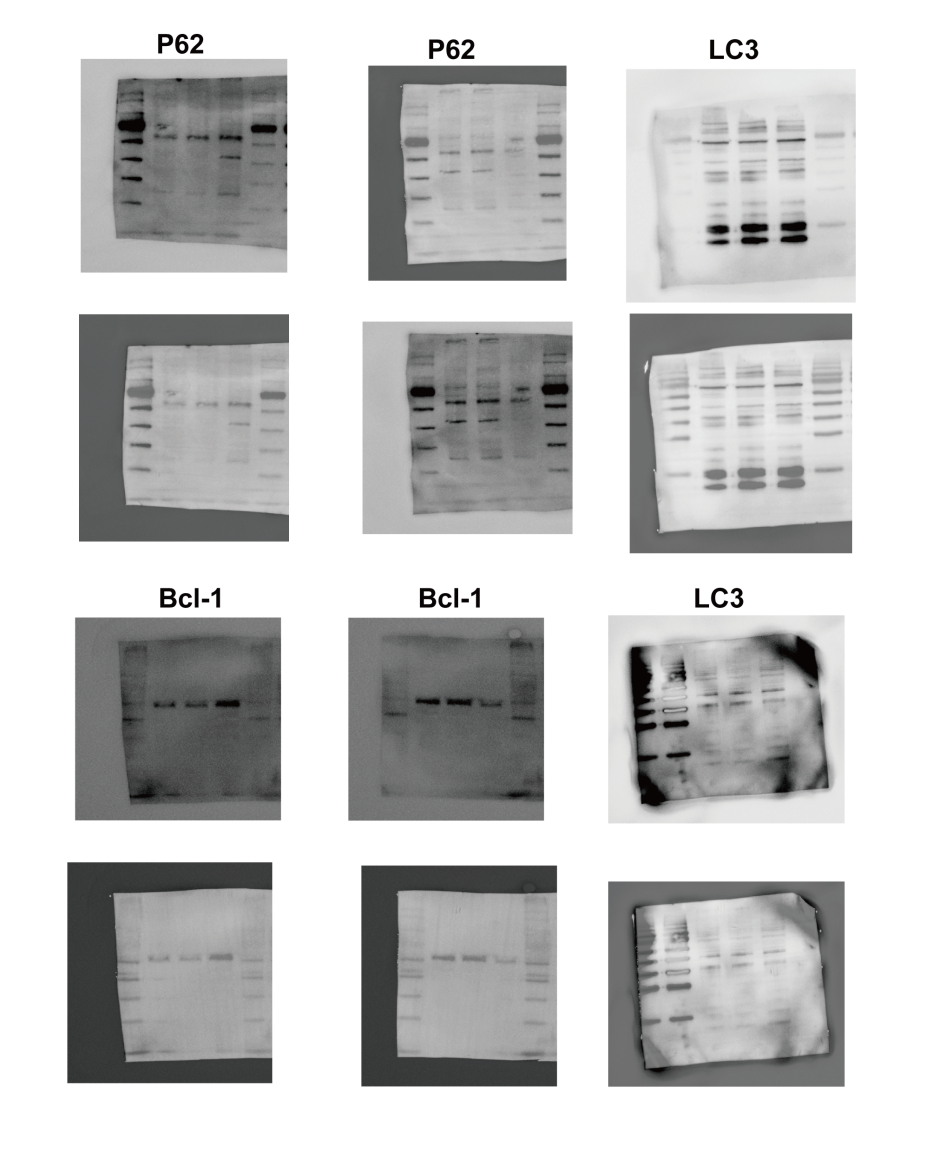


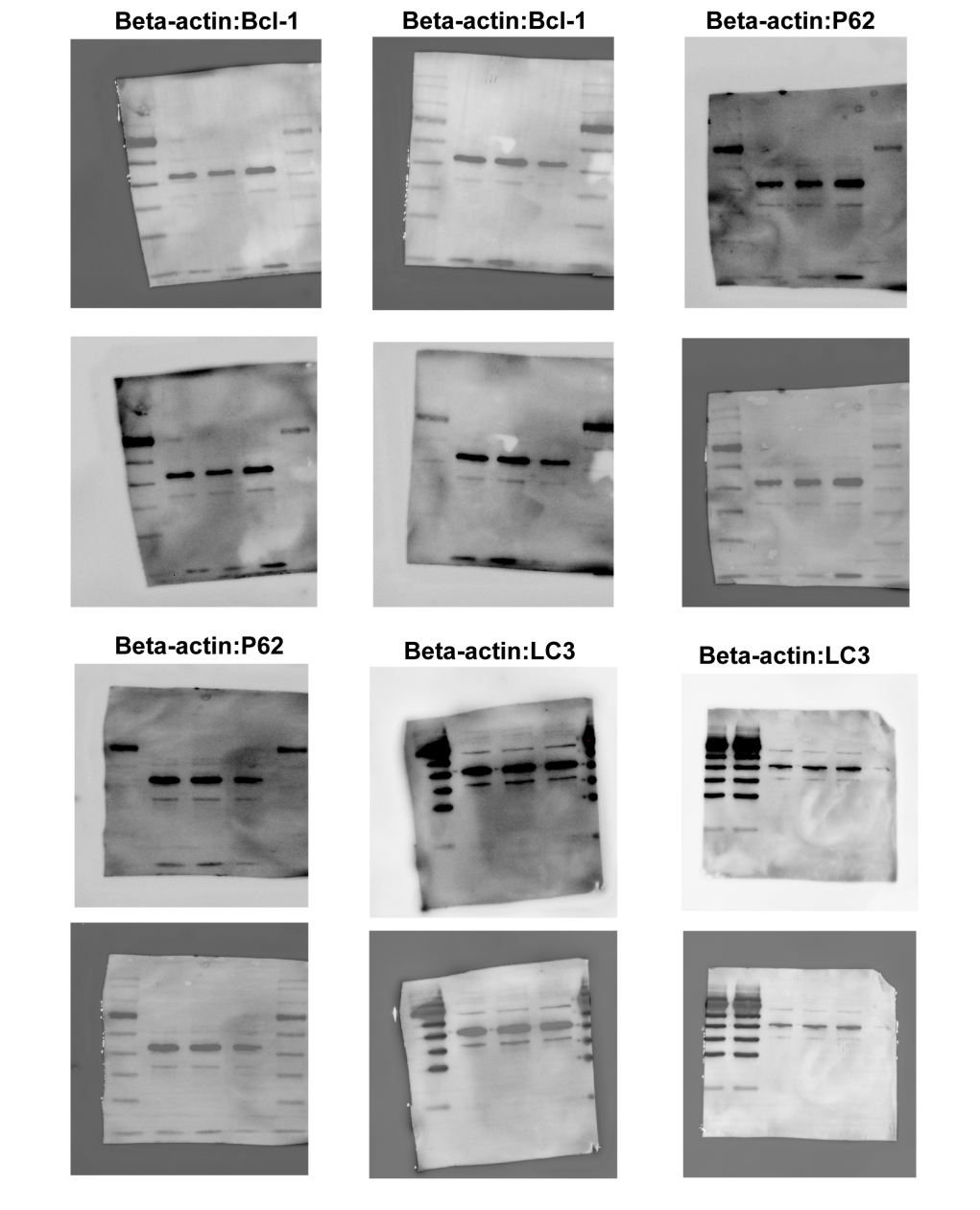

Supplement: Supplementary file 2 [file DataSheet1.docx]
